# Preparation, Optimization and Characterization of Chitosan-coated Liposomes for Solubility Enhancement of Furosemide: A Model BCS IV Drug

**DOI:** 10.22037/ijpr.2019.111834.13384

**Published:** 2020

**Authors:** Mohammad Moslehi, S. Ali Reza Mortazavi, Amir Azadi, Samaneh Fateh, Mehrdad Hamidi, S. Mohsen Foroutan

**Affiliations:** a *Department of Pharmaceutics, School of Pharmacy, Shahid Beheshti University of medical Sciences, Tehran, Iran. *; b *Department of Pharmaceutics, School of Pharmacy, Shiraz University of medical Sciences, Shiraz, Iran. *; c *Department of Pharmaceutics, School of Pharmacy, Zanjan University of medical Sciences, Zanjan, Iran.*; Most of the drugs are administered orally and they must be absorbed from gastrointestinal tract for systemic effect. Generally, drug molecules get dissolved in GI fluids before absorption. Solubility and permeability of drugs are considered as two parameters in biopharmaceutical classification system (BCS). Many researches were performed to change permeability and solubility of the drugs to improve abruption and bioavailability of them. Lipidic nanocarriers such as nanoliposomes are one approach to dissolve poorly water soluble drugs. Furosemide (FMD) as a typical molecule in BCS IV has low solubility and permeability. Thermodynamically, molecules of FMD could stay in the space of liposomal membrane. In this study effective parameters to preparation of nanoliposomes coated by chitosan (CCLs) were screened optimized by experimental design. Particle size, polydispersity index, and surface potential of optimized CCLs have 155.8 ± 3.5 nm, 0.229 ± 0.022, and 25.2 ± 3.5 mV, respectively. FMD was loaded in optimized CCLs 98.94 ± 0.7%. Size of CCLs have verified by atomic force electron microscope. CCLs loaded by FMD were dried and *in-vitro* study was performed to test release of FMD from powder. Results demonstrated that solubility and dissolution of FMD increased by loading in CCLs in comparison to crystalline FMD and physical mixture of FMD and materials of CCLs.

**Keywords:** Chitosan, Optimization, Liposome, Furosemide, Solubility

## Introduction

Oral drug delivery is the most common route for delivery of pharmaceuticals and other bioactive agents. This route of administration can be used both for local (i.e., for GI disorders) or systemic purposes. For both of these intentions, the drug molecule should be dissolved in gastrointestinal fluids adequately. In this context, solubility and permeability of the drugs have been suggested as two important factors determining the final oral bioavailability of the drug molecules; a concept nowadays formulated as the biopharmaceutical classification system generally referred to as BCS (1). Several well-designed studies have been reported on these two parameters (2-6) and the relationship of the *in-vitro* dissolution and the GI absorption of the different drugs (7, 8). Poorly water soluble drugs, consisting of more than 70% of the therapeutic molecules, have a serious challenge with their solubility in body fluids, thus suffering from low as well as erratic oral bioavailability problem against their successful development and clinical use (9, 10). Dissolution rate of the drugs, on the other hand, is dependent on the aqueous solubility, which causes another problem against the bioavailability. There is a general belief that an aqueous solubility less than 100 mg/mL in aqueous will be resulted to absorption problem based on dissolution (11). To solve the poor solubility problem, different approaches have been attempted such as particle size reduction of the active pharmaceutical ingredients (12), complexation, e.g., inclusion complex (13, 14), changing the crystalline structures to amorphous state (15-19), and salt forming (20, 21) among others. Using the nanocarriers, mainly including nanoparticles and nanovesicles are efficient approach highly attractive for solubility enhancement (22). The two core concepts behind this application is increasing the total surface area of the drug in contact to the solvent as well as the conversion of the drug crystalline form to the amorphous state (23). Nanocrystals (24), nanoemulsions (25-27), solid lipid nanoparticles (28) and nanoliposomes (29, 30) are among the mostly studied systems in this context. 

Liposomes, firstly discovered and reported by Bingham (31), are vesicles made of phospholipid bilayers using different techniques such as thin-film hydration (32, 33), reverse-phase evaporation (34), dehydration-rehydration (35, 36), alcohol injection (37), micro fluidization (38), and inverted emulsion (39). Liposomes are regularly used as carriers for drug delivery purposes. Their use for some other purposes has also been attempted, among them the application of these vesicles as solubility/stability enhancer being of interest regarding the scope of the current study (40).

Composition or method of preparation is proven to effect the type and size of liposomes. Thin-film hydration method usually make multi lamellar vesicles (MLV) with large and heterogeneous sizes. Liposome preparation by this method usually needs extrusion or sonication for size reduction of vesicles (41). Ethanol injection method is a one-step method that commonly makes small and homogenous vesicles (37).

Liposomes could be prepared and applied as the naked or surface-treated liposomes. In the latter case the treatment is mainly done using polymers (42). The majority of phospholipids used to liposome preparation cause negative surface charge, which is a basis for surface treatment by polycationic compounds via the electrostatic attractions. Chitosan is a cationic polymer derived from the naturally occurring chitin which has attracted recent interests for this purpose to form nanostructures known as ‘chitosomes’. These modified liposomes seem to be more stable, less negatively charged, and capable of bioadhesion to the biological epithelia. 

In this study, furosemide (FMD), a model BCS class IV drug, i.e., suffering both from low water solubility and low membrane permeability was selected as a candidate for solubility improvement using a nanoliposome formulation surface-treated by chitosan. 

## Experimental

*Materials*

Chitosan (medium molecular weight) was purchased from Sigma-Aldrich (Cat No.: 448877, St. Louis, USA). Phosphatidylcholine E80 (from Egg-yolk) was purchased from lipoid® GmbH (Ludwigshafen, Germany). Furosemide was kindly donated by Caspian-Tamin Co. (Tehran, Iran). All other materials and solvents used throughout the study were of analytical or HPLC grade, whenever needed, and were purchased locally.

*Preparation of chitosan-coated liposomes (CCLs)*

CCLs were prepared based on ethanol injection method. For this purpose, phosphatidylcholine (PC) was dissolved in ethanol to make the organic phase which was, then, added dropwise to 50 mL distilled water as the aqueous phase while being stirred magnetically, using a precise injection pump (Mindray, SK-500 II) working with a precision of 0.1 mL/min. After liposome preparation, the resulting dispersion was stirred in room temperature for an additional 40 minutes to stabilize the vesicles. 

After completion of the liposomes preparation, 30 mL chitosan solution in acetate buffer (0.67 M of sodium acetate trihydrate; different pH values) was injected into the liposome dispersion while being stirred magnetically. The obtained solution was finally stirred for 40 min in order to accomplish the electrostatic attraction between negative surface changes of liposomes and positive charges of chitosan molecules. The set of parameter values were optimized as described later in the article. 

*CCLs optimization*

A statistical factorial design was used for the optimization of the preparation process for CCLs. Based on our preliminary experiments and the similar studies, firstly eight parameters were selected for screening step, including phosphatidylcholine amount, ethanol/water ratio, injection rate of organic phase, stirring rate of water for liposome preparation, water temperature, concentration of chitosan in buffer, pH of buffer and rate of addition of chitosan solution to liposome dispersion (Table 1).

Particle size (z-average), poly disparity index (PDI) and surface potential were used as responses of the study. These eight factors at two levels designed according to Plackett–Burman method. 12 experiments were designed and carried out to screening effective factors. In the next step, effective factors in three levels (Table2) were designed for optimization according to D-Optimal method. 

Based on this method, 25 experiments were arranged and their effects on responses were studied. Obtained data were evaluated and fitted to by DesignExpert® software (version 7.0.0). Finally, optimum method for CCL preparation was verified by five additional experiments.

*Furosemide loading in CCLs*

Furosemide (FMD) is a lipophilic drug (logp of 2). Therefore, it was dissolved in ethanol with phosphatidylcholine before being incorporated in CCLs. For this purpose, 23.7 mg of FMD was dissolved in ethanol concurrent to the phospholipid. The solution was, then, injected into the stirred water under the condition specified earlier for the CCLs preparation based on the statistically optimized set of the parameters.

To determine the loading parameters of the drug in nanoliposomes, the dispersion of CCLs in water was centrifuged (Hettich, Rotina 380R, Germany) at 15000 g for 15 minutes at 25 °C using the commercially available micro filtration tubes (Sartorius, 100,000 D). The filtrate was analyzed for unloaded drug concentration spectrophotometrically at 275 nm (Reyleigh UV-2601). Another sample was prepared according to CCLs preparation method but without FMD to be used as matrix blank for UV analysis.

The efficiency of encapsulation (EE%) of the drug was calculated as percentage of loaded amount of FMD to the total amount added during the loading procedure. 

$\mathrm{EE\%}=\frac{\mathrm{Encapsulated Drug}}{\mathrm{Total Drug}}=\frac{\mathrm{Total FMD}-\mathrm{Free FMD}}{\mathrm{Total FMD}}\times 100$ (1)

Also loading capacity (LC%) of FMD in CCLs loaded by FMD was calculated by below equation.

LC%=FMDEncap.CCL total (2)

Where, FMD_Encap_ is the total amount of the added drug multiplied by the EE% and CCL_total_ is the total weight of drug-loaded carrier including phospholipid, chitosan, and the loaded drug.

*Characterization of FMD-loaded CCLs*

*Particle size and surface potential analysis*

The size as well surface potential of the nanocarriers were monitored during the optimization as well as upon the re-validation of the finally selected form using dynamic light scattering method (DLS) by 90° scattering light angle and a He/Ne laser light (λ=633 nm) (Malvern Instruments Ltd., model Nano ZS, UK). The samples with and without drug loading were diluted similarly by water in 25 °C and DLS study was carried out in triplicate.

*Atomic force microscopy image*

The size and topology of the bare liposomes, CCLs, and dried-reconstituted CCLs were tested by Atomic Force Microscopy (AFM) (JPK, model NanoWizard II, Berlin, Germany). 

 Aqueous suspensions of each type was air-dried using the standard technique before the imaging procedure.

*Physical stability of CCLs*

CCLs loaded by FMD were prepared using the optimized condition and placed in ambient temperature (25 °C). To ensure the short-term stability of the vesicles before drying, the samples of CCLs loaded by FMD were analyzed for average size, polydispersity index, and surface potential in 24, 48, and 72 h after preparation.

*In* *-* *vitro release study*

The *in-vitro* release profile of the drug out of the carriers was evaluated using USP dissolution apparatus II (Erweka-DT 820) in two steps. Firstly, release of FMD was evaluated in simulated gastric fluid (SGF) for 2 h, then, followed by the simulated intestinal fluid (SIF) for 8 h. SGF was prepared according to United States Pharmacopeia (USP) by dissolving 2.0 g of sodium chloride in 7.0 mL of hydrochloric acid and sufficient water to make 1000 mL. Also SIF was prepared by dissolving 6.8 g of monobasic potassium phosphate in 250 mL of water. It was mixed with 77 mL of 0.2 N sodium hydroxide. The pH of resulting solution adjust with 0.2 N sodium hydroxide or 0.2 N hydrochloric acid to a pH of 6.8 ± 0.1 and then was diluted with water to 1000 mL. The release media were stirred at 50 rpm in 37 °C ± 0.5 °C throughout the study. Three samples were taken at 0.5, 1, and 2 h from dissolution vessels (Figure 5) and filtered by Polyvinylidene Fluoride (PVDF,0.22 µm) before the drug analysis followed by sampling and drug assay at 5, 10, 20, 30, 60, 120, 240, and 480 minutes after the start of the step 2. Release profiles of FMD were studied in three groups including crystalline powder of FMD, CCLs loaded by FMD, and the physical mixture of FMD with drug-free CCLs. All tests were performed in triplicate. The samples of both steps were analyzed by high performance liquid chromatography (HPLC) as described later in this article. 

*Characterization of CCLs in dried form*

Drying of liposomes is an approach for stability increasing of the carriers. In this study, spray-drying was applied for drying the carriers. Suspension of FMD-loaded CCLs was dried by Nano-spray-dryer (BUCHI, model B-90 HP, Switzerland). The inlet temperature of spraying was set on 100 °C with the outlet temperature being 29 °C. The inlet air compressor was set at 24 mbar. CCLs loaded with FMD and without FMD were dried in the similar condition. To evaluate the possible effect of the drying process on the stability of the carriers a sample of the dried CCLs was reconstituted by dispersing the precise amount in distilled water in such a way that the total solid amount in this suspension was similar to CCLs suspension before drying. The reconstituted suspension was evaluated for average size, polydispersity index, and surface potential using the dynamic light scattering (DLS) method. The result of this test was compared to the CCLs samples before drying.

*Differential scanning calorimetry (DSC)*

Thermal analysis was performed by using DSC (Mettler Toledo, model DSC 823, Greifensee, Switzerland) on solid materials and nanopowder of FMD-loaded CCLs. Approximately 9 mg of powder was placed in aluminum crucible and one empty crucible was employed as reference. Thermal analysis was carried out from 20 °C to 400 °C range and at a constant rate of 10 °C/min. 

*FMD assay*

For measurement of FMD concentrations in different samples during the study, a 1 mL aliquot of the nanodispersion was dissolved in ethanol (1 to 20 dilution) and was stirred 60 minutes for disrupting the membrane. The resulting solution was, then, filtered for injection into HPLC.

HPLC analysis was performed using a reversed-phase system. The HPLC system (Knauer, model Smartline, Germany) with a PDA detector (Knauer, model 2800, Germany) was used for the analysis. As stationary phase, a C8 column (150 mm × 4.6 mm, 5 µm; Knauer, Eurospher II, Berlin, Germany) with the same guard packing was used. The mobile phase consisted of water: acetonitrile: acetic acid (75:75:1). It was filtrated and then degassed by vacuum and sonication. The flow rate was 1 mL/min at 25 °C temperature. The content of FMD in injected amount of samples was determined by UV detection at 234 nm wavelength. 

## Results

*Factorial design*

CCLs were prepared by method described generally earlier in this article. Eight different factors tested primarily for their effect on the overall procedure (Table 1). The relative effects of these factors on three selected responses (i.e. size, PDI and zeta potential) were studied using the standard Plackett–Burman method. Results showed that 4 parameters had no significant effect on responses, including P.C amount, stirring rate of water phase for initial liposome preparation, concentration of chitosan in buffer, and additional rate of chitosan solution onto liposome dispersion. Optimization of CCLs was, then, attempted by D-optimal method and the variation of these factors was studied on responses. (Table 2). 

Based on the two step statistical design, the final optimal set of parameters were selected for CCLs preparation as shown in Table 3.

**Figure 1:**
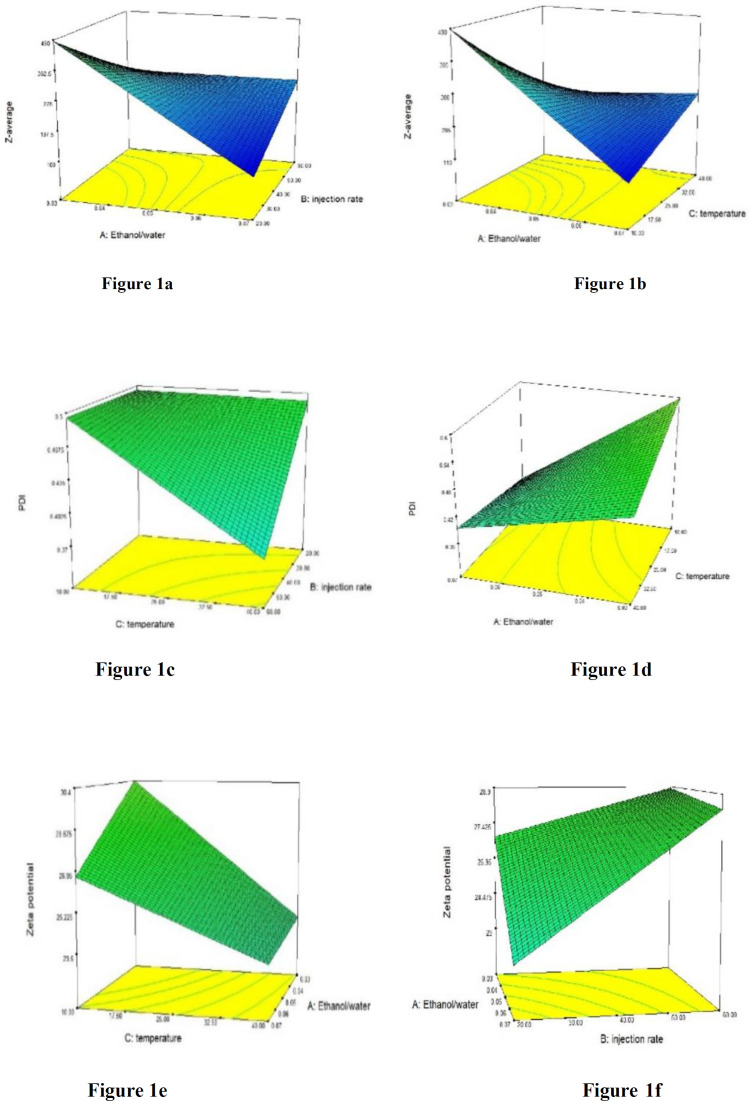
Some 3-D graphs of interaction between effective parameters

The graph of interaction between some parameters have been shown in Figure 1 (a-f). 

**Figure 2:**
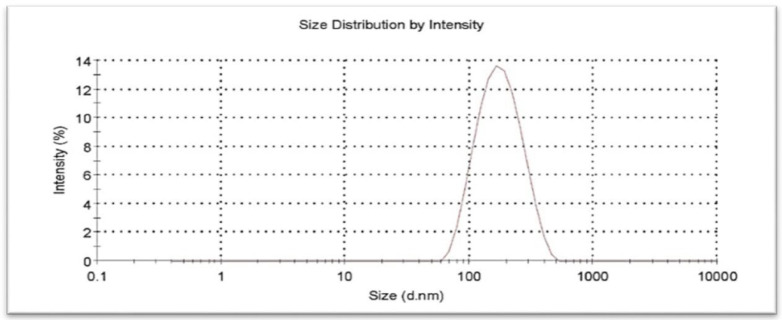
Size distribution of optimized chitosan coated liposome analyzed by DLS

Five verification runs confirmed final formulation and lack of fit of model was 0.16 which, therefore, was identified as non-significant. Also average size, PDI, and zeta potential of the optimally prepared CCLs were 155.6 ± 3.5 nm, 0.229 ± 0.022, and +25.2 ± 3.5 mV, respectively. A typical particle size distribution curve of the optimized nanocarriers is shown in Figure 2.

Saesoo *et al.* (43) prepared liposomes with phosphatidylcholine by thin film hydration for loading of cisplatin. They applied a quaternized N,O-oleoyl chitosan as a modified chitosan in liposome composition for coating of liposome. Their results showed that liposomes without coating have 141.7±1.7 nm diameter. Liposome formulation with coating increased diameter to 171.5 ± 0.8 nm. However loading of cispelatin in coated liposome with N,O-oleoyl chitosan formed vesicles with 164.6 ± 1.0 nm diameter. 

Also our results showed that loading of FMD in chitosan coated liposomes did not change size of vesicles. 

*Loading parameters of FMD in CCLs*

**Figure 3:**
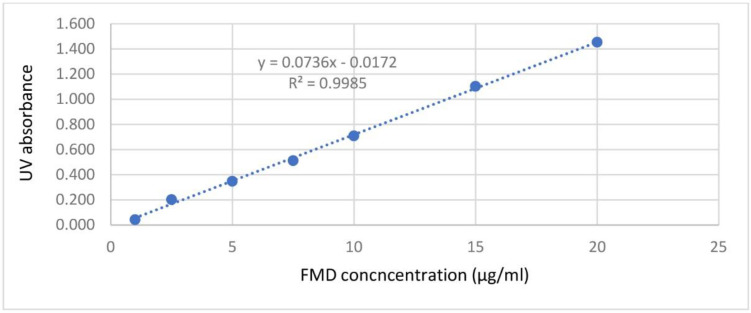
Calibration curve of FMD in filtrates passed through the microfiltration device, analyzed by UV-spectrophotometry

After optimization of CCLs, as described, FMD was loaded in the nanocarriers. For this purpose, 23.7 mg of FMD was dissolved in ethanol together with P.C and was injected into the distilled water. FMD molecules were hypothetically localized within the phospholipid bilayer thickness in liposomes. After completion of CCLs preparation upon the chitosan coating, 5 mL of CCLs loaded by FMD was centrifuged within microfiltration tubes (Sartorius, Vivaspin®6, Germany) and the filtrate was, then, analyzed spectrophotometrically at 235mm to determine the FMD concentration. The results showed that 98.94 ± 0.7 % of the used FMD was loaded in CCLs (EE%). Calibration curve of FMD by UV-spectrophotometry was constructed between 1 µg/mL to 20 µg/mL as showed in Figure 3.

Theoretically, poorly water-soluble molecules were localized in the membrane of liposome while staying between lipophilic chains of phospholipids. The efficiency of loading of such molecules in liposome membrane could be affected by the characteristics of the molecule and its interaction with the membrane; among the effective factors the lipophilicity of the molecule, changing of the membrane polarity by the molecule, and the steric fitness of molecule the between membranes can be listed briefly (44). Moreover, study of Tan *et al.* (45) demonstrated that coating of liposome by biopolymers could increase the loading of molecules in liposomal bilayer. They found that chitosan coated on liposome could inhibit the leakage of carotenoids. In other study, Zhou *et al*. (46) used chitosan-coated liposomes for loading of acteoside. They found that liposome coating caused increased loading efficiency of acteoside from 81% to 88%. 

Furosemide is a small molecule with log P of 2 which defines it as a hydrophobic compound. It, therefore, tends to stay within bilayer of liposomes, as described. Additionally, it has a carboxylic acid group with pKa of 3.9. Thus, in low pH condition, higher fraction of the drug molecules become non-ionized and, therefore, show more hydrophobic characteristics. Therefore, high yield of encapsulation of FMD in CCLs was predicted considering the mild acidic pH of the chitosan solution. 

Also total weight of FMD which was dissolved and injected by ethanol is 23.7 mg. As EE% of FMD that is said above, encapsulated amount of FMD in CCLs is 23.45 mg. On the other hand, total weight of CCL with loaded FMD is 473.45 mg and so LC% of FMD is 4.9%. Palazzo *et al*. (47) have been studied EE% and LC% of estradiol liposome and drug in cyclodextrin in liposome (DCL). They prepared liposome using 1palmitoyl 2 oleoylsn glycero 3 phosphocholine (POPC) by lipid film hydration method. They found that both EE% and LC% depend on the initial percentage of estradiol to POPC. Higher amount of this ratio has been caused higher LC% as 1.6% and 3.8% respectively for liposome and DCL and lower initial ratio has been caused lower LC% as 1.0% and 2.1%, respectively for liposome and DCL. 

*Drying of the nanocarriers*

The aqueous dispersions of the CCLs both with and without FMD were dried by nano-spray-dryer. The dried powder was collected and based on the input mass, the final yield of the process was calculated using the following equation: 

Yield %=Final mass of CCLsTotal input mass of process solids×100 (3)

The results showed that yield of drying for FMD-loaded CCLs and drug-free CCLs were 38.3% and 41.4%, respectively.

Similar spray-dryer was used to drying solution containing Arabic gum, maltodextrin, polyvinyl alcohol, and modified starch and drying yield were 74.7%, 43.0%, 80.9%, and 94.5%, respectively (48). 

A number of other studies have reported the spray drying for preparation of liposomal powders. Lo *et al*. (49) prepared superoxide dismutase (SOD) loaded in liposomes made by four types of phospholipids, with the final sizes of 150 to 200 nm. They mixed liposomes with sucrose, lactose, or trehalose as stabilizing agents in spray drying process. Their results showed that the activity of SOD was preserved, but liposomal vesicles were wrinkled upon drying. They used liposome vesicles as naked and without any coating. In the other study Chen *et al*. (50) spray-dried liposomes loaded by bovine serum albumin (BSA) and coated by alginate, chitosan and trimethyl chitosan (TMC). Their result showed that BSA was protected in sphere-like dried vesicles. 

In the other study researchers dried chitosan-coated liposomes loaded by ghrelin using spray dryer (51). They tried to optimize four parameters of spray dryer to obtain the best vehicles. They found that regarding the vehicle size, gas flow had significant reverse effect and feed rate also had significant reverse effect on total weight of the collected dried powder. Various sets of the drying condition resulted in 7.5% to 87.4% (w/w) final yield. Their results showed that z-average and PDI of the vesicles increased upon drying from 195 ± 6 and 0.082 to 263 ± 5 and 0.203, respectively.

*Particle analysis of CCLs with atomic force microscopy (AFM)*

**Figure 4:**
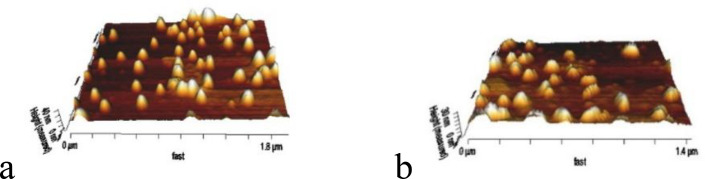
AFM images of FMD-Loaded CCLs (a) and FMD non-loaded CCLs (b).

Dried powder of CCLs with and without loading by FMD were analyzed by AFM. Image of AFM showed that size and topography of CCLs are similar in FMD loaded and non-loaded vesicles (Figure 4). The regular vesicular shape, smooth surfaces, and the sizes of the carriers confirmed our findings as well as basic knowledge from the CCLs prepared. 

*Differential scanning calorimetry*

Figure 5 shows results of thermal analysis by DSC. Thermograph of crystalline powder of FMD shows a sharp exothermic peak in 220 °C and an endothermic peak in 282 °C (a). Some other studies on FMD show these peaks (52, 53). These peaks also is in mixture of FMD, phosphatidylcholine, and chitosan (b). This thermogram shows an endothermic peak near to 50 °C. According to some studies on structured, formed by phospholipids, thermograph of bilayer membrane shows endothermic peaks about 30 °C to 60 °C. Temperature point of this peak could be related to lipid chain of applied phospholipid (54, 55). The endothermic peak which is related to bilayer structure clearly is in thermograph of CCL loaded by FMD. It seems that endothermic peak in 282 °C is related to decomposition of FMD. It is in thermogram of pure FMD, mixture, and CCLs loaded with FMD.

*FMD Assay in dried CCLs powder*

Amount of FMD was determined in FMD-loaded CCLs before and after drying. Firstly FMD was analyzed in CCLs dispersion before drying. As described, the dispersion was dissolved in ethanol and the concentration was analyzed by HPLC. The amount of FMD in non-dried CCLs was 99.21% of the predicted amount. The FMD amount was analyzed in dried CCLs, to ensure the drug chemical stability upon spray drying, showing a ratio between the dried to non-dried vehicles of 101.22%. Therefore, it was evidenced that FMD was completely stable against the spray drying procedure. 

Vanic *et al.* (56) prepared tablets of pre-liposomes containing metronidazole in their study. They prepared pre-liposomes from metronidazole, lecithin and mannitol by spray drying and used different excipients for making tablets. They dissolved Pre-liposome powders in methanol for evaluating drug content after spray drying. They considered dried powder characteristics, mechanical tablet properties, drug release, and liposomal characteristics. They found that excipients of tablets effect on drug release from liposomes which make will be made *in-situ*.

*Stability of CCls*

CCLs loaded by FMD and optimized for size, PDI, and surface potential were studied for physical stability of the nanodispersion. The results showed that CCLs were stable upon storage in room temperature for a reasonable time before drying (Table 5).

*In-vitro drug release*

As mentioned, three samples were prepared for the *in-vitro* study: CCLs loaded by FMD, the free crystalline powder of FMD, and the physical mixture of the crystalline FMD, and the materials used in preparation of the drug-free CCLs. As shown in Figure 6, after 120 minutes in simulated gastric fluid (SGF) none of the samples could release FMD for more than 10%. But the CCLs released is FMD more than the other types (8.1 ± 0.6 %) and the statistical calculations showed that dissolution of FMD from CCLs in SGF is significantly higher than crystalline FMD and physical mixture (P-value ˂ 0.001) after 2 h. In simulated intestinal fluid (SIF), however, three samples could release the whole FMD contents in relatively short time periods, with all of them approaching the 100% plateau in the first 60 minutes (Figure 7). As shown in Figure 7, the rate of FMD dissolution from CCLs was remarkably higher than the others. CCLs showed more than 96 ± 3.3% drug in 5 minutes, whiles in the similar time period the dissolved drug from the crystalline powder and the physical mixture were 42.3 ± 1.9% and 56.6 ± 3.0, respectively. The statistical calculations showed that dissolution of FMD from CCLs in SIF is significantly higher than crystalline FMD (*P*-value ˂ 0.001) and physical mixture of FMD with other materials (*P*-value ˂ 0.001) after 5 minutes.

Radwan *et al.* (57) prepared nanoparticles by chitosan and alginate and loaded them by FMD with a final size of about 250 nm. They studied FMD dissolution from those carriers in HCL (0.1 N) and phosphate buffer (pH=5.8). Obviously, FMD was dissolved by higher rate and the amount of nanoparticles compared to the free FMD in HCL medium. However, the difference of drug dissolution profile from nanoparticles and the free drug was less in phosphate buffer compared to the acidic condition. In the other study, Shariare *et al.* (53) prepared nanosuspension of FMD with 119.1±6.0 nm particle size and compared FMD dissolution profile from the nanosuspensions and free FMD in gastric pH (pH=1.2). The results indicated more than 80% of FMD dissolution in 5 min from the nanosuspension whereas no discernible amount of FMD was dissolved within 60 min from the free drug.

**Figure 5a:**
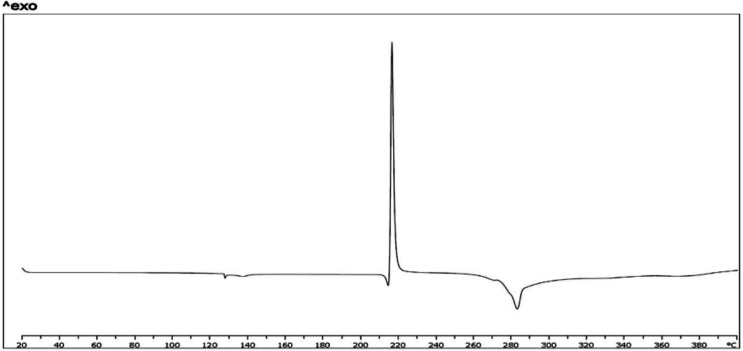
(FMD)

**Figure 5b:**
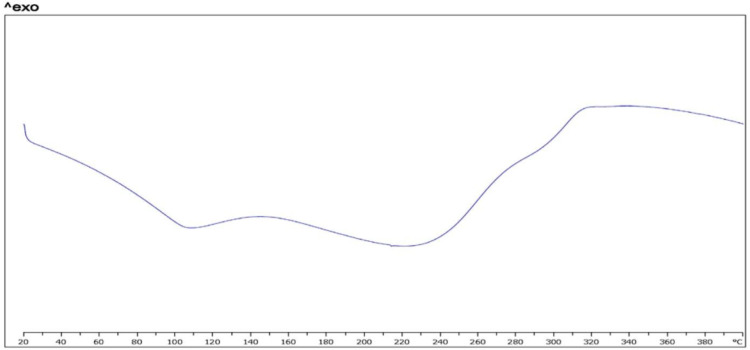
(chitosan)

**Figure 5c:**
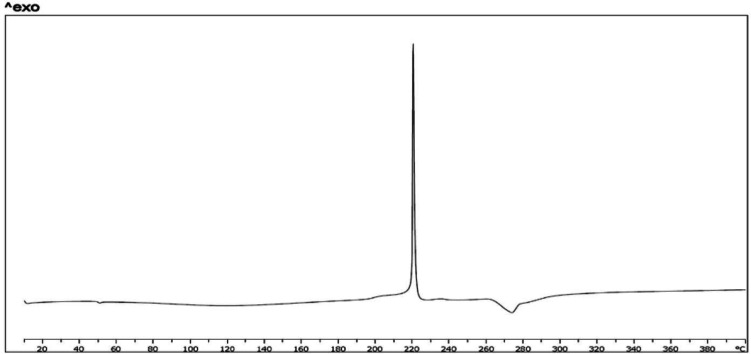
(physical mixture)

**Figure 5d:**
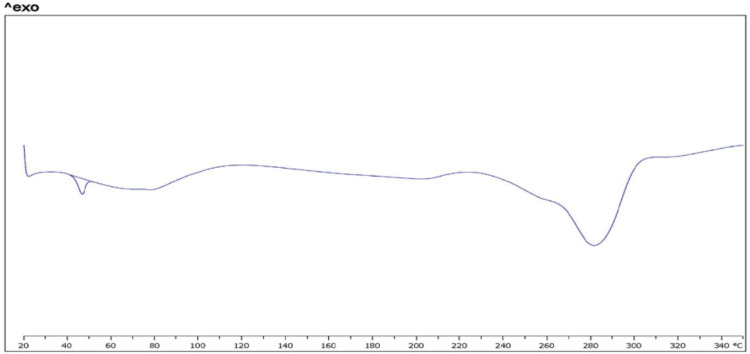
(nanopowder of CCLs loaded FMD)

**Figure 6:**
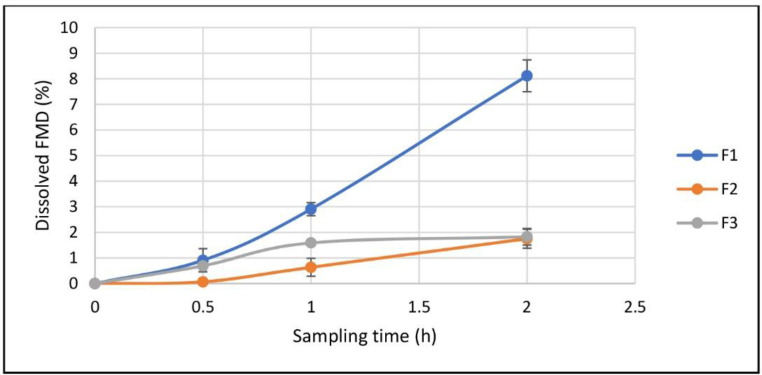
Dissolution of FMD in simulated gastric fluid from dried drug-loaded CCLs (F1), physical mixture of FMD CCLs materials (F2), and free crystalline FMD (F3), (Mean ± SD, n = 3).

**Figure 7.**
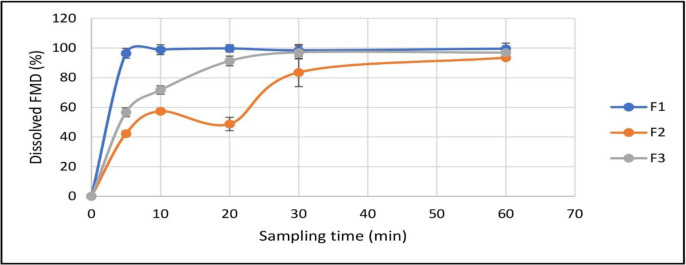
Dissolution of FMD in simulated intestinal fluid from dried drug-loaded CCLs (F1), physical mixture of FMD CCLs materials (F2), and free crystalline FMD (F3), (Mean ± SD, n = 3).

**Table 1:** Parameters selected for screening in CCLs preparation procedure

**No**	**Factor**	**Unit**	**Lower level**	**Upper level**
1	Phosphatidylcholine amount	mg	0.2	0.4
2	Ethanol/water ratio		0.025	0.075
3	Ethanol injection rate	mL/hr	20	60
4	Stirring rate	RPM	800	1800
5	Temperature	°C	10	40
6	Chitosan concentration	% (w/v)	0.25	0.75
7	pH of chitosan buffer		4.1	5.5
8	Chitosan solution injection rate	mL/hr	20	60

**Table 2:** Effective parameters and their levels for CCLs optimization

**No**	**Factor**	**Unit**	**Lower level**	**Middle level**	**Upper level**
1	Ethanol/water ratio		0.025	0.05	0.075
2	Ethanol injection rate	mL/hr	20	40	60
3	Temperature	°C	10	25	40
4	pH of chitosan buffer		4.1	4.8	5.5

**Table 3:** Effective parameters and actual values for optimized CCLs preparation

	**Parameters**	**Actual value**
A	Ethanol/water volume ratio	0.06
B	Ethanol injection rate	37.39 mL/hr
C	Temperature	40 °C
D	pH	4.22

**Table 4:** Characteristics of CCLs with and without FMD-loaded

	**without FMD**	**with FMD**
Particle size (nm)	155.6±3.5	152±2.2
PDI	0.229±0.022	0.234±0.029
Surface potential (mv)	25.2±3.5	27.4±2.9

**Table 5:** Characteristics of nanoparticles with and without Chitosan-Coating

	**Naked Liposomes**	**Coated Liposomes**
Particle size (nm)	127.4±3.3	155.6±3.5
PDI	0.231±0.013	0.229±0.022
Surface potential (mv)	-9.5±2.9	25.2±3.5

**Table 6:** Stability of CCLs loaded by FMD

	**0 h**	**24 h**	**72 h**
Particle size (nm)	154.5	157.8	153.1
PDI	0.219	0.234	0.229
Surface potential (mv)	+23.9	+25.3	+25.7

## Conclusion

In this study, chitosan-coated liposomes, sometimes referred to as ‘chitosomes’ in literature, were prepared by the simple and available ethanol injection method using egg phosphatidylcholine and, then, were coated by medium molecular weight chitosan based on electrostatic attraction between the negative charge of liposomes and positive charge of chitosan. The prepared nanocariers were, then, successfully optimized statistically in terms of the three responses of size, PDI, and zeta potential. Thereupon, furosemide as a practically insoluble drug molecule from BCS IV was loaded in liposomes at the same time of liposome preparation and it is predictable that it was ‘intercalated’ within the liposomal bi-layer wall. The vesicles were successfully dried by the spray drying process using a professional nano-spray-dryer; however, a significant amount of CCLs was lost during the drying. The dried nanocarriers showed ideal size, shape, and stability with the drug dissolution profile being remarkably improved both in simulated gastric and intestinal fluid as the main finding we expected from this project. The impact of this improvement in solubility and dissolution rate on the oral bioavailability of this model BCS IV drug is under investigation in our laboratory.
